# Empirical comparison of structure-based pathway methods

**DOI:** 10.1093/bib/bbv049

**Published:** 2015-07-21

**Authors:** Maria K. Jaakkola, Laura L. Elo

**Keywords:** pathway analysis, pathway structure, comparison, functional genomics, statistical analysis

## Abstract

Multiple methods have been proposed to estimate pathway activities from expression profiles, and yet, there is not enough information available about the performance of those methods. This makes selection of a suitable tool for pathway analysis difficult. Although methods based on simple gene lists have remained the most common approach, various methods that also consider pathway structure have emerged. To provide practical insight about the performance of both list-based and structure-based methods, we tested six different approaches to estimate pathway activities in two different case study settings of different characteristics. The first case study setting involved six renal cell cancer data sets, and the differences between expression profiles of case and control samples were relatively big. The second case study setting involved four type 1 diabetes data sets, and the profiles of case and control samples were more similar to each other. In general, there were marked differences in the outcomes of the different pathway tools even with the same input data. In the cancer studies, the results of a tested method were typically consistent across the different data sets, yet different between the methods. In the more challenging diabetes studies, almost all the tested methods detected as significant only few pathways if any.

## Introduction

In the past years, pathway analysis has become a common operation in functional genomics studies, as investigating single gene activities alone has turned out not to be sufficient. Multiple tools for pathway analysis have been proposed, making the selection of a suitable tool difficult. Most of the tools simply consider the pathways as unstructured gene sets, and define the pathway activity as enrichment of the pathway genes among the top detections [1–4]. There are numerous reviews and comparison studies investigating the performance of these tools [5–7]. Much less studied are pathway tools that take into account the pathway structure when determining pathway activities. There is a recent comparison of tools using pathway structure [8], but only in theoretical level. In another study [9], some of the tools were tested but not systematically compared. So far, the structure-based methods have not reached the popularity of the unstructured methods, and there is lack of knowledge on their actual utility in practice. Therefore, this study aims at providing such knowledge in a systematic way. We briefly review mathematical algorithms behind six different pathway analysis methods and compare the tools based on them at an empirical level. The practical usability of a tool is important for an average final user who is likely to apply it with the default settings. To maximize comparability of the different methods, all preprocessing steps and cutoff values are fixed to be the same if the method does not explicitly tell otherwise. Also, KEGG pathways [10] used for testing are chosen so that all the methods can analyze them. The methods were chosen based on their mathematical basis to represent clearly different approaches, as well as their availability and functionality. The two last criteria dropped out, surprisingly, many method candidates. Methods to be tested were signaling pathway impact analysis (SPIA) [11], centrality-based pathway enrichment (CePa) [12], network-based gene set analysis (NetGSA) [13], functional annotation tool of database for annotation, visualization and integrated discovery (DAVID) [6, 14], gene set enrichment analysis (GSEA) [15] and Pathifier [16].

## Pathway methods

In this section, we shortly describe the mathematical basis of each of the six tested methods. Some basic information about features and versions of the methods applied in this study is presented in Table 1. More detailed information about the methods can be found from the original articles. Although the methods have different mathematical basis behind them, they share some notation. Let us denote by *G*, a pathway that consists of *p* genes $g_{1},g_{2},..,g_{p}$. Analogously, by $A=\{s_{1},s_{2},\ldots ,s_{m}\}$, we denote a set of *m* samples including a subset of case samples *A_C_* and a subset of control samples *A_R_*. Genes are indexed with *i* and samples with *j*. The term DE genes refers to differentially expressed genes.

**Table 1.** General information about the tested pathway analysis methods

Software	Input from user	Output for each pathway	Version	Reference
Methods using pathway structure				
SPIA	DE genes with values; background genes; pathway files from KEGG [10]	FDR	2.18.0	[10]
CePa	DE genes; background genes	Multiple *P*-values	0.5	[11]
NetGSA	Gene expression matrix; sample labels; pathway structure	*P*-value	1.0	[12]
Methods not using pathway structure				
DAVID	DE genes; background genes	FDR	6.7	[6, 13]
GSEA	Gene expression matrix; sample labels; gene sets	FDR	build 0039	[14]
Pathifier	Gene expression matrix; sample labels; gene sets	FDR for each sample	1.4.0	[15]

All information in the table is about those versions of the methods used in this study. Most of the methods can use different types of input data, and the output might include additional information not listed here.

### Methods using pathway structure

#### SPIA

In SPIA, the score for pathway *G* is defined as

SSPIA(G)=PNDEPPB−PNDEPPB ln(PNDEPPB),

where *P_NDE_* is the probability that the pathway includes at least the observed number of DE genes when the null hypothesis is true, and *P_PB_* is the probability that the pathway has at least as high total perturbation as observed (assuming again null hypothesis). The null hypothesis for *P_NDE_* is that all DE genes are distributed randomly in a list of measured genes, and for *P_PB_* that the pathway DE genes take random places in the pathway. Details about the calculation of *P_NDE_* and *P_PB_* are provided in the original publication [11]. Total perturbation $PB_{tot}^{obs}$ of the pathway *G* is calculated as a sum of the accumulated perturbations of the genes in the pathway:

(1) $$PB_{tot}^{obs}(G)=\sum_{i\in G}\left(PF(i)-\Delta D(i)\right),$$

where ΔD(i) refers to the expression change of gene *i* (log fold-change ratio). The term *PF*(*i*) corresponds to the perturbation of gene *i*, including both measured perturbation and perturbation inherited from its parent nodes and is defined as

(2) $$PF(i)=\Delta D(i)+\sum_{k\in parent(i)}\beta_{ki}\cdot \frac{PF(k)}{n_{child}(k)},$$

where *parent*(*i*) refers to parent nodes of gene *i*, and $n_{child}(k)$ is the number of child nodes of gene *k*. Coefficient *β_ki_* tells the type of interaction between parent *k* and child *i* (1 for activation and −1 for inhibition).

#### CePa

The centrality-based pathway enrichment tool CePa includes multiple different ways to consider pathway structure [12]. In this study, we concentrate on an overrepresentation analysis (ORA) extension because of its ability to handle missing measurements in an expression data set. In the ORA extension of CePa, the final pathway score of pathway *G* is defined as

(3) $$S_{CePa}(G)=\sum_{i\in G}centrality(i)\cdot de(i),$$

where

$$de(i)=\{\begin{array}{cc} 1, & \text{if}\;\text{gene}\;i\;\text{is}\;\text{differentially}\;\text{expressed}\\ 0, & \text{otherwise}. \end{array}$$

The term *centrality*(*i*) can have different definitions, and it is up to the user to choose which one to use. It can be, for example, the length of the longest shortest path to leaf node, the length of the longest shortest path to root node, the number of child nodes or the number of parent nodes. To avoid favoring pathways with certain structure (e.g. chain-like pathways), multiple centrality criteria are used.

#### NetGSA

The mathematical model behind NetGSA is rather complex, so we describe here only its general concept. More detailed explanation is available in the original papers [13, 17]. The expression profile vector *e_j_* of sample *j* consists of real signal and noise and can be defined as

(4) $$e_{j}=\Lambda \gamma_{j}+\epsilon_{j},$$

where $\epsilon_{j}$ corresponds to the noise. The real signal $\Lambda \gamma_{j}$ consists of the individual effect of each gene and influence of other genes. The coefficient vector *γ_j_* is a latent variable representing the individual effect. The matrix Λ is a weighted influence matrix that contains the information about the relations between the measured genes. The NetGSA test statistic for pathway *G* is then defined as

$$S_{NetGSA}(G)=b_{G}\cdot X_{C}-b_{G}\cdot X_{R},$$

where vector *b_G_* indicates which genes belong to pathway *G* and *X_C_* and *X_R_* are matrices including vectors *e_j_* as columns, where *j* belongs to case samples *A_C_* and control samples *A_R_*, respectively. Testing the null hypothesis $H_{0}:E(S_{NetGSA}(G))=0$ against the alternative hypothesis $H_{1}:E(S_{NetGSA}(G))\neq 0$ is done by implementing the latent variable model (4) as a mixed integer model.

### Methods not using pathway structure

#### DAVID

The DAVID tool is based on modified Fisher’s exact test. In the basic Fisher’s exact test, genes are divided into two groups based on two criteria: whether a gene is DE, and whether it belongs to a specific pathway. Then the probability of having a given number of DE genes in a pathway is calculated using hypergeometric distribution. DAVID uses Fisher’s exact test with jackknifing [18, 19]. That means that, one gene is repeatedly removed from the group of DE genes that belong to a pathway under consideration and then the probability is calculated. This aims to eliminate pathways whose significance is strongly dependent on only few genes that might be false-positive DE genes.

#### GSEA

The first step in GSEA is to form a decreasing ranked list, which consists of all the *n* genes in the data. In a typical case, the ranking of a gene *i* is done according to differential expression *d*(*i*) between two groups of samples, for example, healthy and sick. After the ranked list of genes has been formed, an output value *S_GSEA_* can be calculated for each pathway (gene set) *G*. The score $S_{GSEA}(G)$ is defined as the maximum difference between 0 and *a cumulative sum*, which can be formulated as

(5) $$S_{GSEA}(G)=\underset{r}{\max}|P_{hit}(G,r)-P_{miss}(G,r)|,$$

where $P_{hit}(G,r)$ corresponds to genes in the ranked list belonging to pathway gene set *G* up to a given rank *r*, and $P_{miss}(G,r)$ those genes that do not belong to *G*. The term $P_{hit}(G,r)$ is defined as

$$P_{hit}(G,r)=\sum_{\begin{array}{l} i\in G\\ r(i)<r \end{array}}\frac{|d(i){|}^{u}}{\sum_{i\in G}|d(i){|}^{u}},$$

where *d*(*i*) is an estimate of differential expression of gene *i*, *r*(*i*) is the rank of gene *i* and *u* can have different values. The most common choices for *u* are *u* = 0 and *u* = 1. The term $P_{miss}(G,r)$ is defined as

$$P_{miss}(G,r)=\sum_{\begin{array}{l} i\notin G\\ r(i)<r \end{array}}\frac{1}{n-p},$$

where *p* is the number of genes in pathway gene set *G*. The significance *P*-value of $S_{GSEA}(G)$ is calculated by randomly permuting the sample labels and computing $S_{GSEA}(G)$ for that case. This process is repeated 1000 times.

#### Pathifier

Unlike other methods considered here, the Pathifier tool calculates a score *S_Pathifier_* for each sample $s_{j}\in A$ and every pathway *G*. When analyzing pathway *G*, only gene expression measurements of genes belonging into *G* are considered. Now all the samples can be reduced to vectors of length *p*, where *p* is number of genes in pathway *G*. The score is based on nonlinear principal curve [20] generated from all the reduced samples $A^{*}$. After finding the principal curve, the score for sample *s_j_* and pathway *G* is the distance between the projection of the reduced sample $s_{j}^{*}$ and the projection of a centroid of the reduced normal samples $cent(A_{R}^{*})$ along the curve. Let function arch(x,y,z) denote the distance between *x* and *y* along the curve *z*. Now Pathifier score $S_{Pathifier}(G,s_{j})$ can be formulated as

(6) $$S_{Pathifier}(G,s_{j})=arch(projec(s_{j}^{*}),projec(cent(A_{R}^{*})),pc(A^{*})),$$

where *pc* is the principal curve and function *projec* returns the projection of a particular sample to the principal curve $pc(A^{*})$.

## Comparison design

### Data set preprocessing and methods to detect DE genes

Because SPIA, CePa and DAVID need a list of DE genes as input, we used two tools to find the DE genes: Limma (version 3.22.4) [21] and ROTS (version 1.1.1) [22, 23]. Limma was chosen because of its popularity and ROTS because it has performed well in previous comparison studies [22, 23]. To compare the sample-level Pathifier results with the results from the other pathway methods, we transformed the sample-specific results into group-level results between case and control samples using Limma and ROTS.

All data sets were tested as unscaled measurements and as base-two logarithm–scaled measurements. Before logarithm transformation, a constant one was added to all measurements to prevent negative values without causing big changes in the general range of measured values. If the authors of a method have advised that the method should be used with scaled or unscaled input data, this advice was respected. Otherwise both preprocessing approaches were considered. GSEA takes as input unscaled data, and Pathifier and NetGSA take as input logarithm–scaled data. For the methods that use as input lists of DE genes (SPIA, CePa and DAVID), there were no such recommendations.

In the comparisons, the cutoff value for significant pathways was defined so that false discovery rate (FDR) was <0.05. If a method returns only *P*-values, they were converted to FDR values using the Benjamini–Hochberg method. With CePa, a pathway is considered as significant if it has FDR value < 0.05 according to at least one of six ready-made ways to define the centralities.

To have comparable results from different methods, the same pathways should be analyzed. We used KEGG database [10] as a pathway source because all the methods can process that pathway format. After excluding pathways that can not be analyzed by all the six methods, 86 KEGG pathways remained for the analyses.

### Test design

To evaluate the different pathway methods, multiple data sets from two different conditions were considered, including six data sets on clear cell renal cell carcinoma (ccRCC) and four data sets on type 1 diabetes (T1D). A method is considered as reliable if it returns consistent results from similar study settings. Results are expected to vary to some extent because of individual differences and differences in study designs such as measurement protocol, number of samples and the type of control samples (e.g. paired or not). To compare different methods, we developed a scoring method based on weighted number of pathways found from multiple data sets scaled by the average number of false positives. The score is defined as

(7) $$score(method)=\frac{1000}{T}\cdot \sum_{h=\left\lceil l/2\right\rceil }^{l}\frac{\beta (method,h)\cdot {\left(h-\left\lceil l/2\right\rceil +1\right)}^{2}}{{\left(\alpha (method)+1\right)}^{2}},$$

where the parameter *l* describes the total number of tested data sets and it is six for ccRCC and four for T1D, and ⌈⌉ denotes the ceiling function, which returns the smallest integer greater or equal to a given number. Function *β* tells how many pathways a given *method* found from exactly *h* data sets. Function *α* tells how many pathways the given *method* found in artificial data sets on average (false positives). Constant 1 is added to the denominator to avoid dividing by 0. The scaling term *T* is the theoretical maximum score, which depends on the number of tested pathways and used data sets. With 86 pathways and six data sets (ccRCC tests), *T* gets a value of $86\cdot {\left(6-3+1\right)}^{2}=1376$, and with 86 pathways and four data sets (T1D tests), it is $86\cdot {\left(4-2+1\right)}^{2}=774$. This term is included into the *score* to make ccRCC and T1D test scores comparable. Because in practice, the *scores* are much lower than the theoretical maximum, a coefficient 1000 is included to keep the general level of *scores* in a readable level. The score is calculated separately for each method with scaled and original data and with Limma and ROTS, if applicable.

### Data sets

Data from two different diseases representing different characteristics were used to test and compare the pathway methods. In ccRCC data sets, individuals are heterogeneous, but changes between sick and healthy samples are relatively big. In a more difficult case of T1D, the data are heterogeneous and differences between the sample groups are relatively small. This causes ccRCC data sets to include typically thousands of DE genes, whereas <20 genes appear as DE in T1D data sets.

Six ccRCC data sets were downloaded from the GEO database [24] and they are identified as GSE781, GSE11024, GSE14762, GSE14994, GSE6344 and GSE15641. Four data sets related to T1D were downloaded from two sources, GEO [24] and ArrayExpress [25]. T1D data sets GSE9006, GSE30211 and GSE51058 are from GEO, and data set TABM-666 is from ArryExpress. Information about data sets, platforms and arrays is presented in Table 2. Some of the data sets included measurements from multiple arrays, but only the measurements from the arrays listed in Table 2 were used in this study.

**Table 2.** Information about the data sets used for comparing the pathway methods

Data set id	Data base	Platform	Array	Number of samples case + control	Number of genes
Clear cell renal cell carcinoma data sets					
GSE781	GEO	Affymetrix	HG-U133A	9 + 8	12 752
GSE6344	GEO	Affymetrix	HG-U133A	10 + 10	12 752
GSE15641	GEO	Affymetrix	HG-U133A	32 + 23	12 752
GSE14994	GEO	Affymetrix	HG-U133A	22 + 8	12 743
GSE11024	GEO	Affymetrix	HG-U133 Plus 2.0	10 + 12	17 699
GSE14762	GEO	Affymetrix	HG-U133 Plus 2.0	10 + 12	17 232
Type 1 diabetes data sets					
GSE9006	GEO	Affymetrix	HG-U133A	19 + 24	12 752
GSE30211	GEO	Affymetrix	HG-U219	13 + 12	19 040
GSE51058	GEO	Illumina	HumanHT-12	21 + 15	17 981
TABM666	ArrayExpress	Affymetrix	HG-U133 Plus 2.0	3 + 3	20 156

If a ccRCC data set included samples other than ccRCC or healthy controls, they were left out from analysis. With T1D data sets, the selection of samples to be used was more complicated because the T1D data sets often included time series measurements or matched control samples. From T1D data set GSE9006, we compared the healthy control samples with samples taken from T1D patients 4 months after T1D diagnosis. From data sets GSE30211 and TABM-666, we compared newly diagnosed T1D samples at the time of diagnosis and strictly matched control samples, matched by age, gender, date of birth and genetic risk. In data set GSE51058, we compared individuals who developed T1D with those who did not, using measurements at seroconversion to autoantibody positivity.

The microarray data sets included original probes/probesets instead of genes and they needed to be transformed before pathway analysis. In case one probe/probeset referred to multiple genes, it was removed from the analysis. In case multiple probes/probesets referred to one gene, just one of them was selected based on the highest variance among all the samples.

Besides the 10 real comparisons (six in ccRCC and four in T1D), artificial data sets were also tested. The purpose of these artificial data sets was to find out if the methods favor some type of pathways or find plenty of noise pathways in general. The artificial data sets were generated by picking all controls from one T1D and one ccRCC data set and randomly dividing them into artificial case and control groups. Five unscaled and five logarithm-scaled data sets were generated from the ccRCC data set, and five unscaled and five scaled data sets were generated from the T1D data set.

## Results

**Figure 1.**
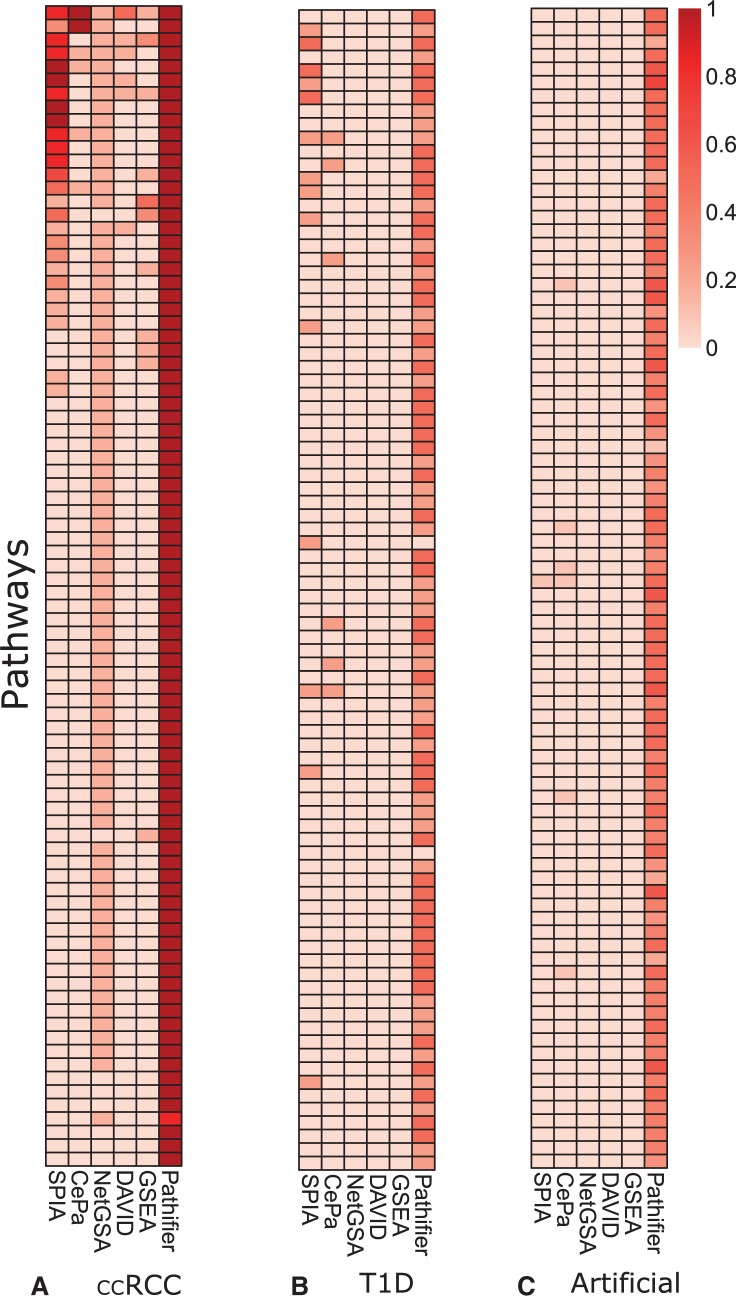
Consistency of the significant pathways (rows) identified using the six different tools (columns) in (**A**) six ccRCC data sets, (**B**) four T1D data sets and (**C**) 10 artificial data sets. The heatmap illustrates the percentages of data sets in which each pathway (row) is detected as significant. In artificial data sets, no consistent findings are expected. A colour version of this figure is available at BIB online: http://bib.oxfordjournals.org.

In this section, we describe our results for the tested pathway methods in the 10 ccRCC or T1D data sets, as well as additional 10 artificial data sets where no significant pathways are expected. To ensure comparability between the computational methods, we focus on pathways supported by all of the tools. In the subsection ‘Comparison between ROTS and Limma’, we compare results from Limma and ROTS, otherwise we concentrate on results obtained when the list of DE genes was determined by ROTS. A general overview of the consistency of the results is presented in a heatmap format in Figure 1.

### Consistency of ccRCC results

**Figure 2.**
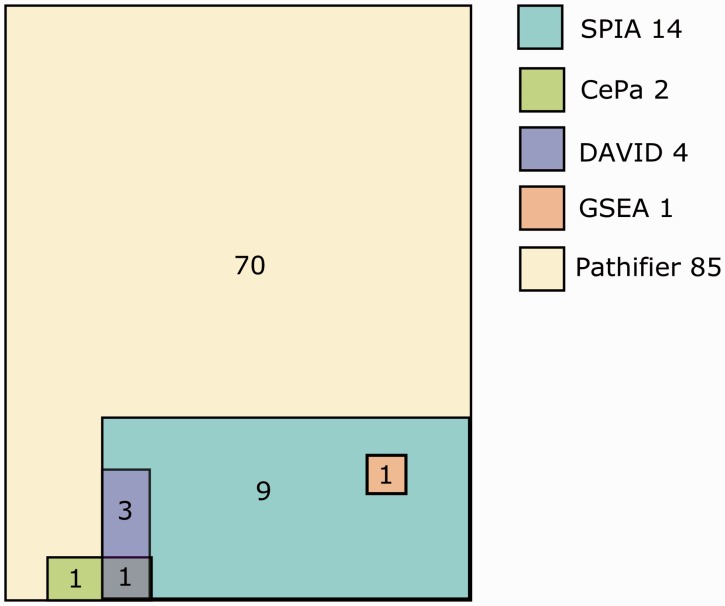
Number of overlapping pathways detected by different methods from ccRCC data set GSE14994. NetGSA results were excluded because it did not detect any pathways significant from that data set. The total numbers of detected significant pathways are indicated after the method names. In total, 86 pathways were tested. A colour version of this figureis available at BIB online: http://bib.oxfordjournals.org.

In general, the results from the different methods were not similar in the six tested different ccRCC data sets (Figure 1A). Significant pathways according to different methods were different, and the number of significant findings was more dependent on the method than on the data set. Overlap of significant pathways detected by different methods is illustrated in Figure 2.

SPIA found rather many significant pathways from the ccRCC data sets. Although there were several pathways found from only one data set, in general, results from SPIA were consistent. From scaled ccRCC data sets, 11 pathways were detected consistently from five or six of the six ccRCC data sets. Results from scaled and unscaled data sets were not identical, but the general trends were mostly similar. From artificial data sets, SPIA detected only one false-positive significant pathway from one data set.

Scaling had rather big effects on the CePa results. Without scaling, the results were not consistent between different data sets. With logarithm-scaled data sets, two pathways were marked as significant for all data sets. These pathways were PPAR signaling pathway (KEGG accession hsa03320) and leukocyte transendothelial migration (KEGG accession hsa04670). Findings from artificial data sets were few and not consistent.

With NetGSA, one data set (GSE15641) had almost all pathways detected as significant; yet there were not any significant findings from the other data sets. With the other methods, the contrast between GSE15641 and other data sets was not apparent. This indicates that with NetGSA, cutoff values should be carefully adjusted for the data set under consideration. From artificial data sets, NetGSA did not find any pathways significant.

GSEA and DAVID made few significant findings in our comparisons and pathway selection. None of the pathways was found in more than four data sets by DAVID and in more than three data sets by GSEA. GSEA was tested only with unscaled data sets because of the author’s recommendations, but DAVID was tested with both scaled and unscaled data sets. From unscaled data sets, DAVID found more significant pathways than from scaled data sets. Both DAVID and GSEA detected few, if any, pathways significant from artificial data sets.

Pathifier results with different data sets were consistent, but not informative. Data set GSE14994 had one pathway not detected as significant, otherwise all pathways from all the data sets were significant. Based on the author’s recommendation, only scaled data sets were tested. There were also more findings from artificial data sets compared with the other methods. There is one interesting side about Pathifier results with artificial data. Results from the artificial data sets generated based on controls from T1D data set are greatly different from those from ccRCC-based artificial data. From the ccRCC-based data, few noise pathways were detected, whereas from the T1D-based artificial data sets, plenty of significant pathways were found, which are considered as false positives. Such phenomena did not appear with the other tested pathway methods.

**Figure 3.**
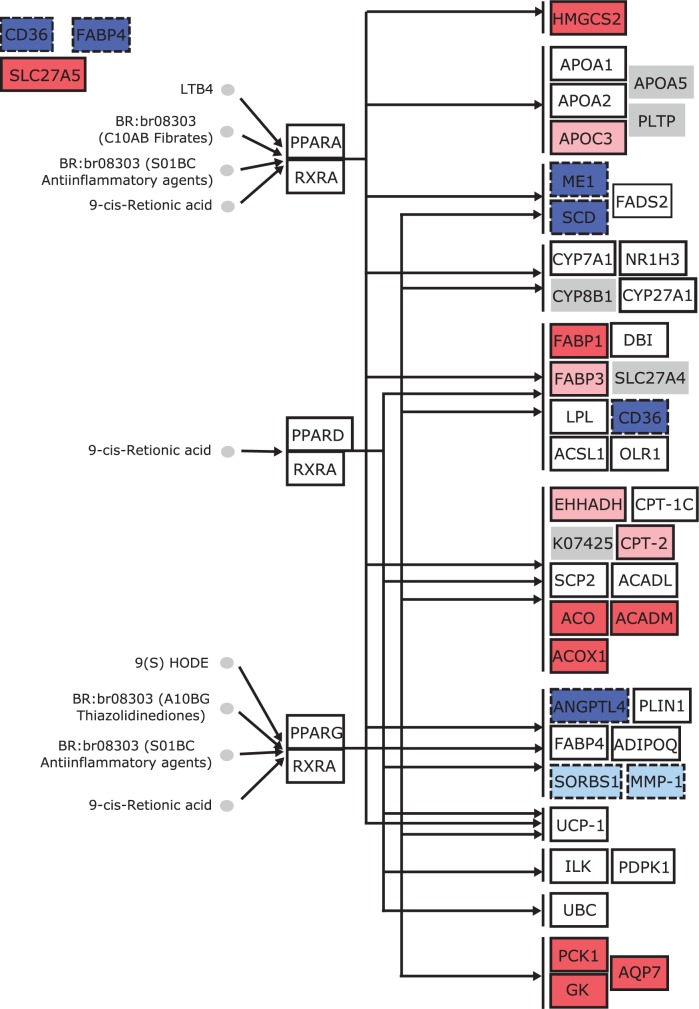
PPAR signaling pathway from KEGG. The nodes correspond to genes and other functional units, and edges represent interactions between those units. Nodes are colored based on their differential expression between case and control samples detected with ROTS in the ccRCC data set GSE14994. Solid borders indicate that the gene is highly expressed in ccRCC patient samples, and correspondingly dashed borders mean low expression compared with control samples. If FDR value of the node is ≤0.05, the color of the node is strong. The color is light if the FDR value is between 0.05 and 0.1. Genes with white node color have FDR value >0.1. For gray nodes without borders, there are no measurements available. A colour version of this figure is available at BIB online: http://bib.oxfordjournals.org.

Table 3 shows the pathways that appeared significant in at least four data sets according to at least one method, excluding Pathifier, which detected basically all the pathways in each data set. Figure 3 illustrates the only pathway, PPAR signaling pathway, that satisfied the selection criterion by two methods (SPIA and CePa) in addition to Pathifier. The pathway has been linked to ccRCC in literature [26, 27].

**Table 3.** Pathways found as significant from at least four ccRCC data sets by at least one method, excluding Pathifier

Pathway	SPIA	CePa	NetGSA	DAVID	GSEA	Pathifier
PPAR signaling pathway	0.83 (0.00)	1.00 (0.00)	0.17 (0.00)	0.50 (0.00)	0.17 (0.00)	1.00 (0.50)
Cytokine–cytokine receptor interaction	0.83 (0.00)	0.00 (0.00)	0.17 (0.00)	0.00 (0.00)	0.00 (0.00)	1.00 (0.50)
ECM–receptor interaction	1.00 (0.00)	0.00 (0.00)	0.17 (0.00)	0.00 (0.00)	0.00 (0.00)	1.00 (0.50)
Complement and coagulation cascades	0.83 (0.00)	0.17 (0.00)	0.17 (0.00)	0.17 (0.00)	0.00 (0.00)	1.00 (0.50)
Leukocyte transendothelial migration	0.33 (0.00)	1.00 (0.00)	0.17 (0.00)	0.00 (0.00)	0.00 (0.00)	1.00 (0.50)
Intestinal immune network for IgA production	0.67 (0.00)	0.00 (0.00)	0.17 (0.00)	0.00 (0.00)	0.17 (0.00)	1.00 (0.20)
Allograft rejection	0.83 (0.00)	0.00 (0.00)	0.17 (0.00)	0.17 (0.00)	0.33 (0.00)	1.00 (0.20)
Viral myocarditis	0.83 (0.00)	0.00 (0.00)	0.17 (0.00)	0.17 (0.00)	0.17 (0.00)	1.00 (0.50)
Systemic lupus erythematosus	1.00 (0.00)	0.00 (0.00)	0.17 (0.00)	0.17 (0.00)	0.00 (0.00)	1.00 (0.70)
Natural killer cell-mediated cytotoxicity	0.83 (0.00)	0.17 (0.00)	0.17 (0.00)	0.00 (0.00)	0.00 (0.00)	1.00 (0.50)
Antigen processing and presentation	1.00 (0.00)	0.17 (0.00)	0.17 (0.00)	0.00 (0.00)	0.00 (0.00)	1.00 (0.60)
Focal adhesion	1.00 (0.00)	0.00 (0.00)	0.17 (0.00)	0.00 (0.00)	0.00 (0.00)	1.00 (0.50)
Chemokine signaling pathway	0.83 (0.00)	0.00 (0.00)	0.17 (0.00)	0.00 (0.00)	0.00 (0.00)	1.00 (0.50)

Columns are methods and each cell describes the proportion of data sets in which the method found the pathway significant. The percentage greater than or equal to 0.67 that brought the pathway to this table is underlined. The corresponding percentages from artificial data sets are shown in parenthesis.

To sum up, in our tests with ccRCC data sets, two methods, SPIA and CePa, had the best balance between consistency and the amount of results. For consistent CePa results, scaling of the data was necessary. Other four methods detected typically few or many pathways significant with the test design used here. Complete tables of significant pathways detected from artificial and ccRCC data sets by different methods and preprocessing procedures are available in Supplementary Tables S1 and S2 respectively.

### Consistency of T1D results

In T1D, the differences between individuals were relatively big compared with disease-related differences, which makes the T1D data more challenging to analyze. In line with this, all the methods made fewer findings from the T1D data sets than from the ccRCC data sets (Figure 1B). Especially from the data set GSE51058, all the methods made hardly any findings. Table 4 shows the pathways that appeared significant in at least two T1D data sets according to at least one method, excluding Pathifier.

**Table 4.** Pathways found as significant from at least two T1D data sets by at least one method, excluding Pathifier

Pathway	SPIA	CePa	NetGSA	DAVID	GSEA	Pathifier
Antigen processing and presentation	0.50 (0.00)	0.00 (0.00)	0.00 (0.00)	0.00 (0.00)	0.00 (0.00)	0.50 (0.60)
Allograft rejection	0.50 (0.00)	0.00 (0.00)	0.00 (0.00)	0.00 (0.00)	0.00 (0.00)	0.50 (0.20)
Viral myocarditis	0.50 (0.00)	0.00 (0.00)	0.00 (0.00)	0.00 (0.00)	0.00 (0.00)	0.50 (0.50)

Columns are methods and each cell describes the proportion of data sets in which the method found the pathway significant. The percentage greater than or equal to 0.50 that brought the pathway to this table is underlined. The corresponding percentages from artificial data sets are shown in parenthesis.

The most interesting T1D results were from SPIA when using logarithm-scaled data sets. Three pathways (antigen processing and presentation pathway, allograft rejection pathway and viral myocarditis pathway) were found significant from half of the data sets, but not from the artificial data sets.

CePa found some pathways significant, but those findings were few and not consistent between data sets. Also from artificial data sets, CePa detected only few pathways significant. Findings from T1D data sets and artificial data sets were not similar to each other.

Two of the methods not using pathway topology (DAVID and GSEA) and one topology-using method (NetGSA) did not find anything from the challenging T1D data sets. These methods had also few, if any, false-positive findings from artificial data sets.

Pathifier again showed more findings than the other methods. Pathifier was not able to analyze the data set TABM-666 because of too few, three, control samples, and from GSE51058 there were otherwise few findings. From the two remaining data sets, there were plenty of significant pathways. Several pathways were repeatedly found also in the artificial data sets.

In general, T1D data sets turned out to be challenging to all the methods. Five of the six tested methods detected few, if any, significant pathways from T1D data sets. The one remaining method, Pathifier, detected around the same amount of significant pathways from T1D data sets and artificial data sets. This makes it difficult to estimate whether Pathifier results are real findings or false positives. A complete table of significant pathways detected from T1D data sets by different methods and preprocessing procedures is available in Supplementary Table S3.

### Comparison between ROTS and Limma

To quantify the consistency of the methods across the six ccRCC data sets or the four T1D data sets, we used the scores defined as in (7). The aim was not to favor either methods with plenty of findings but many false positives or methods with no false positives but only few findings. The scores of the methods with all combinations of scaled and unscaled data sets and ROTS and Limma DE gene detection are presented in Table 5. Complete table of scores of different methods and data sets is available in Supplementary Table S4.

**Table 5.** Scores of the methods describing the amount and consistency of the detections across six clear cell renal cell carcinoma (ccRCC) or four type 1 diabetes (T1D) data sets

Method	Scaled T1D	Original T1D	Scaled ccRCC	Original ccRCC
Scores with ROTS preprocessing				
SPIA	3.20	0.00	79.88	49.42
CePa	0.00	0.00	9.08	0.00
NetGSA	0.00	–	0.00	–
DAVID	0.00	0.00	0.73	3.63
GSEA	–	0.00	–	0.73
Pathifier	0.04	–	0.66	–
Scores with Limma preprocessing				
SPIA	0.00	0.00	47.24	22.22
CePa	0.00	0.00	0.00	0.00
NetGSA	0.00	–	0.00	–
DAVID	0.00	0.00	0.00	0.00
GSEA	–	0.00	–	0.73
Pathifier	0.01	–	1.26	–

The higher the score, the better the method. For NetGSA and GSEA, the method to detect DE genes is not determined by the user, and therefore, NetGSA and GSEA lines are identical in upper (ROTS) and lower (Limma) parts of the table. The dash (–) indicates that the method (row) was not tested in a data set (column).

The highest scores of the methods were typically obtained using ROTS in logarithm-scaled data. If Limma is used to detect DE genes, the results with scaled and unscaled data sets were, in general, closer to each other than the corresponding ROTS-based results. With ROTS, the results were better with scaled input data. The case where preprocessing procedures (scaling and the method to detect DE genes) had most impact on results was ccRCC tests with CePa.

The scores were calculated also for the results from the artificial data sets by dividing the artificial results randomly into two groups of real and artificial results. The scores from artificial results were zero for most of the methods and preprocessing approaches, and they did not exceed 0.005 in any of the cases.

## Discussion and conclusions

In the comparisons, we concentrated on the mathematical algorithms behind the methods and minimized the effect of other properties, such as the selected data base. However, the other ignored features can have a marked effect on the choice of a method or the final results. For instance, for an average user, the ease of use and clear documentation and instructions play a major role when selecting the method.

SPIA was easy to use and the results included information not only about significance of pathway, but also direction of perturbance and size of the tested pathways.

Features of CePa relevant for user are freedom to define which nodes of graph are weighted most. CePa also searches for pathways from multiple data bases and is easy and fast to use. A negative side of the automatic pathway search is that the newest pathways at least in KEGG are not available.

NetGSA results could probably be improved by putting more effort into forming input files. One of the input files is a matrix that includes all known gene interactions. For this study, we used those relations that appear in any pathway, but this could be improved by including also other relations found from literature or other sources. During this study, NetGSA implementation was still under development.

The strength of DAVID is that it is easy and fast to use and does not require heavy preprocessing. It needs only a list of DE genes and even the gene ID type can be any of the common ones. Also, it uses large selection of pathways from different data bases and includes plenty of other functions than pathway analysis as well, such as clustering and gene ID converting.

Like DAVID, GSEA also includes a detailed user manual and it is fast and easy to use. In addition, it can investigate, for example, time series data or data with multiple groups.

Only Pathifier of the six tested methods returns sample-level results. Converting those results to group level with Limma and ROTS and cutoff limit FDR <0.05 provided typically almost all or none of the tested pathways to be significant. This indicates that the cutoff value should be more carefully chosen or that the results should be considered only in sample level.

As expected, all the methods tested found more significant pathways from ccRCC data sets than from T1D data sets. Notably, the structure-based methods (SPIA, CePa and NetGSA) found more results than the non–structure-based ones. This is also a natural outcome because the methods using pathway structure have more input information than the methods based on simple gene lists.

Methods using pathway structure loosely (SPIA and CePa) performed better in our tests than the method using detailed pathway structure (NetGSA). It seems that more complex methods, NetGSA and Pathifier, are sensitive to the set cutoff value because they tend to find either all or none of the pathways as significant in a given data set. In the challenging T1D data sets, none of the tested methods found consistent results. Taken together, our results support the utility of pathway structures in determining pathway activities but also demonstrate the current limitations of the available structure-based tools. In the present comparisons, SPIA showed the best balance between consistency and the number of results and was relatively easy to use.

Key Points

- Pathway methods using pathway topology found more significant pathways than methods not using pathway topology.
- Selection of the method has a large impact on the results.
- With clear cell renal cell carcinoma data, SPIA and CePa provided consistent results with different data sets.
- With type 1 diabetes data, all the methods made only few findings.

## Supplementary Data

Supplementary data are available online at http://bib.oxfordjournals.org/.

Supplementary Data
