# Supplementary material for: Empirical comparison of structure-based pathway methods
Source: Brief Bioinform. 2015 Jul 21;17(2):336–45. doi: 10.1093/bib/bbv049 (PMC4793894; doi:10.1093/bib/bbv049)
Supplement: Supplementary Data [file supp_17_2_336__index.html]

Empirical comparison of structure-based pathway methods — Empirical comparison of structure-based pathway methods — Supplementary Data 

# Empirical comparison of structure-based pathway methods

## Supplementary Data

files

- Supplementary Data - zip file
